# Behavioral responses of domestic cats to human odor

**DOI:** 10.1371/journal.pone.0324016

**Published:** 2025-05-28

**Authors:** Yutaro Miyairi, Yuichi Kimura, Koji Masuda, Hidehiko Uchiyama

**Affiliations:** 1 Department of Animal Science, Graduate School of Agriculture, Tokyo University of Agriculture, Funako, Atsugi, Japan; 2 Department of Animal Science, Faculty of Agriculture, Tokyo University of Agriculture, Funako, Atsugi, Japan; University of Kelaniya, SRI LANKA

## Abstract

People all around the world live with cats and cats engage in many social behaviors toward their owners. Olfaction is one of the most important sensory abilities in cats, yet its role in recognizing humans remains unclear. In this study, we assessed the role and characteristics of olfaction in the discrimination of known or unknown humans by cats using ethological methods. Whether cats exhibit a lateralization of nostril use in response to a variety of olfactory stimuli, exposure experience, *inter alia*, was investigated. Cats were simultaneously presented with three odor stimuli: that of a known person (owner), an unknown person, and a blank control. Responses to the cat 2 scale (Feline Five) and the cat–owner relationship scale (CORS) were collected from cat owners through questionnaires. It was observed that cats spent a substantially longer time sniffing the odor of an unknown person than that of a known person, indicating the use of their sense of smell to distinguish between heterospecific (human) individuals. While responding to odor stimuli from unknown humans, the cats displayed marked lateralization in the use of one nostril or another. An association was observed between the first odor the cat sniffed among known, unknown, and blanks and the personality score. A strong correlation was found between the number of repetitive sniffing odors and personality scores in male cats. No association was evident between the cat’s behavior and the cat–owner relationship score. Rubbing of their faces against an object immediately after sniffing it was observed and thus a possible relationship between the olfactory exploration and subsequent rubbing (odor-marking) behavior in cats is postulated. However, this relationship warrants further investigation along with the theory of whether cats are able to recognize a specific person from olfactory cues.

## Introduction

Dogs (*Canis familiaris*) and cats (*Felis catus*) are popular companion animals that engage in many social behaviors with their owners. Previous studies have focused on the social relationships and behaviors of these animals with their owners. For example, dogs can identify human emotional expressions from facial and vocal information [1], i.e., they can process social information from humans. Cats also have remarkable social cognitive abilities [2], which are gaining the interest of researchers [3]. Studies of cat–human interactions are less advanced than such studies in dogs.

Social cognition is the ability to identify and understand the thoughts, feelings, and perceptions of others [4]. However, many aspects, including fundamental elements such as the perception and cognition of and discrimination between humans (including their owners) by animals are unclear. Social behavior requires the perception of targets, for which animals use sensory systems like vision, audition, and olfaction. Of these, olfaction plays an important role in the social lives of cats [5]. Previous studies have suggested that cats obtain information about health and sexual status (e.g., hormone timing) and known and unknown differences from chemical signals emitted from the urine and feces of similar individuals [6–8]. Furthermore, they exchange odors through allorubbing, i.e., rubbing their bodies against other individuals [9], and the odor marked on a nest by a mother cat has a calming effect on her kittens [8]. Furthermore, cats possess long-term memories of the body odor of their mothers [10] and use volatile components from conspecific anal sac secretions to recognize individuals [11]. These findings indicate that cats use their olfactory senses for social communication and recognition among conspecifics. With regard to human recognition by cats, cats are able to distinguish between humans via their voices [12]. In addition, cats can interpret a human’s gaze to find hidden food [13] and can change their behavior by recognizing emotional states from human odors [14]. However, whether cats utilize their highly-evolved olfactory perception to distinguish between humans has not been studied to date.

Lateralization of brain structure and function has been reported in various animals, and bias in the use of the left and right organs in response to stimuli has been identified. Dogs, domestic chicks (*Gallus gallus*), and horses (*Equus caballus)* preferentially use their right nostril when sniffing novel odor stimuli [15]. Siniscalchi *et al*. show this lateralization in ethological terms as a laterality index [15]. Cats exhibit lateralized ear responses to auditory stimuli, preferentially using their right ears for conspecific voices and their left ears for voices of different species (e.g., dogs) [16]. However, the feline use of the olfactory system remains unclear, including whether cats change their form of nostril use depending on the presence of known and unknown olfactory stimuli. Lateralization and other such characteristics may exist in the feline response to olfactory stimuli, which may include nostril use. The current study aimed to assess the role and characteristics of olfaction in the discrimination of known and unknown humans by cats using ethological methods, viz., comparing the time cats spent sniffing and the number of nostril uses between conditions, and the relationship between each individual’s attributes. Whether cats exhibit a lateralization of nostril use (as in other vertebrates, e.g., dogs) in response to a variety of olfactory stimuli and exposure experience, *inter alia*, was investigated. To test these hypotheses, cats were simultaneously presented with three different odors: those of a known person (the owner), an unknown person, and a blank control. We hypothesized that if cats used olfaction to distinguish between people, they would sniff the unknown odor longer than known odor. We also hypothesized that if cats perform lateralization similar to other vertebrates, such as dogs and horses, they would preferentially use their right nostril for novel stimuli (unknown person’s odor). To test the correlation between the cognitive behavior of cats with humans and personality or depth of relationship with the owner, we collected responses to the cat–owner relationship scale (CORS) [17] and the cat personality scale (Feline Five) [18] from cat owners using questionnaires. This study helps to explain human–cat interactions.

## Materials and methods

### Ethical statement

This study was approved by the Human Ethics Committee (Approval No. 2240) and the Laboratory Animal Ethics Committee (Approval No. 2023050) of the Tokyo University of Agriculture, Japan, with human participation protocols conforming to the Declaration of Helsinki. Informed consent was obtained from the owners via email and in writing. The recruitment of participants started on July 19, 2023 and ended on December 28, 2023.

### Subjects and selection of olfactory stimuli

Thirty domesticated cats were selected for this study, comprising eleven males and nineteen females aged 6.97 ± 0.79 years (mean ± SE), of which twenty-five were spayed and neutered individuals. Seventeen owners of the test animals also participated, consisting of three men and fourteen women aged 41.18 ± 3.19 years (these were the people in the family that took care of the cats the majority of the time). In addition, eight people who had no previous contact with the test animals or their owners and who do not keep any animals were selected to participate as unknown odor donors. Odors were collected from the owners and from unknown persons of the same sex as specific owners to present as test stimuli to the cats. Three odor conditions were established: known odor (owner), unknown odor (non-owner of the same sex as the owner), and blank control (a substrate was used to collect the samples without the addition of human odor).

### Odor collection

A single swab was rubbed in three anatomical areas (five times per area) of each human participant: behind the ear, in the axilla, and between the first and second digits of the foot; this procedure was repeated nine times on the same day, yielding a total of nine swabs per participant. The cotton portions of these swabs were removed, of which three (per participant) were inserted into 1.5 ml sterile and empty tubing. No cross-contamination of the odor from the researcher occurred since the entire process from odor collection to insertion of the cotton portions into the tube was performed by the recipients. For the blank samples, the cotton portion of a clean swab was placed in identical tubing without the prior collection of an odor stimulus. When placing the cotton portion into a tube, the handle of swab after cutting off the cotton portion was used as tweezers to prevent cross-contamination of the odor. To avoid marked variation in body odor due to daily activities, all human participants were asked to refrain from consuming (on the day before sample collection) excessive alcohol, tobacco, spicy food, garlic, and other foods that could affect body odor. The participants were also requested to avoid strenuous exercise, bathing, and the use of perfumes or other strong-smelling products on the day of collection. Both the known odor and unknown odor samples were collected just before the trial.

### Trial apparatus

**Fig 1:**
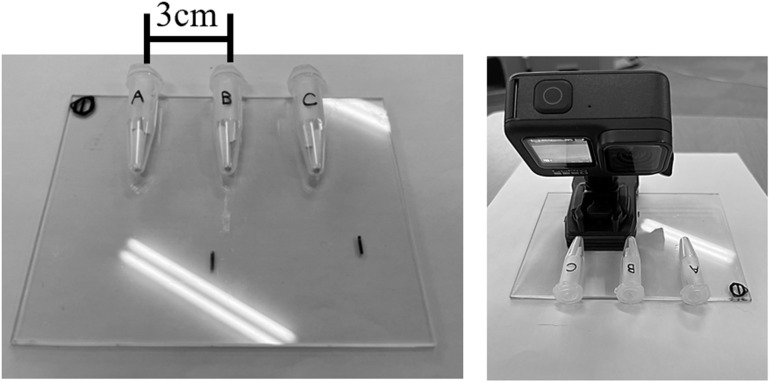
Trial apparatus.

The trial apparatus consisted of 1.5 ml tubes, a transparent acrylic plate (12 cm × 9 cm × 2 mm), and a Mentip hospital cotton swab (No. 1P1504; Japan Cotton Swab Co., Ltd., Japan). A GoPro HERO 9 video camera (GoPro Inc., San Mateo, CA, USA) was used to record the trials (40k30fps). Three tubes containing the three different odor stimuli were fixed to an acrylic plate at three-cm intervals for presentation to a cat: tube A (on the right, from the perspective of the cat), B (in the center), and C (on the left). The video camera was also affixed to the plate (Fig 1).

In this study, the tubes were placed at intervals of 3 cm. This trial setup was selected because of the distance and angle of view was such that the movement of the nostrils could be clearly observed. In addition, we conducted a preliminary trial to confirm whether, during sniffing, the cats place one of their nostrils closer to each tube one by one.

### Behavioral trial

Three trials were conducted with each cat with 5-min intervals between each. Three plates were prepared with three tubes for presentation to a cat. These were prepared in advance and a different plate was presented to the cat for each trial. The order of the three different odor stimuli was randomized in advance: e.g., Trial 1. A = Known, B = Unknown, C = Blank; Trial 2. A = Blank, B = Known, C = Unknown; Trial 3. A = Unknown, B = Blank, C = Known). During each trial, each owner presented the three odor stimuli to the test subject simultaneously. The behavioral response of the cat was recorded until the cat sniffed and eventually left the tubes. In each of the repetitions of the trial, the acrylic plate containing the odor samples was replaced with one of the different arrays, and the video camera was attached anew. The known and unknown odor stimuli were collected just before the start of the trial, and the trial was promptly conducted at each cat’s home. These homes were located within an hour of the odor collection site. In cases where an owner possessed multiple cats and had consented to include all as test animals, the trial procedure was repeated for these animals.

### Questionnaire survey

At the conclusion of the trials, owners completed one online questionnaire per animal tested (S1 Appendix). All owners responded for all the test cats in their house and the number of valid responses was 30. The questionnaire assessed the relationship between the responses of the test animals to odor stimuli and their personalities and the relationships with their owners. Questions were divided into four main categories: (1) questions about the test animals; (2) questions about the owners; (3) a cat–owner relationship scale (CORS), which is a five-point Likert scale comprising scores for three relationship types: perceived emotional closeness, perceived costs, and pet–owner interactions [17]; and (4) the “Feline Five” structure describing cat personality, which is a seven-point Likert scale consisting of scores for five feline personality types: neuroticism, extraversion, dominance, impulsiveness, and agreeableness [18].

### Behavioral and statistical analysis

We quantified the behavioral responses of the cats (such as the time spent sniffing each stimulus type) to different odor stimuli by analyzing the video recordings of the trials. One researcher analyzed the video frame by frame (4k30fps) using the video editing software “Microsoft Clipchamp” (ver. 3.1.12020.0, Microsoft Corporation) and “Bellcurve for Excel” (ver. 3.22, Social Survey Research Information Corporation, Japan). The full definitions of all the measured behavioral patterns is presented in Table 1. The data obtained for different stimulus conditions were compared using the Friedman, Wilcoxon signed-rank, and Kruskal–Wallis tests, as appropriate. The Spearman’s rank correlation coefficient test was used to analyze the relationship between this data and each questionnaire item (e.g., cat personality characteristics). All values are expressed as the mean ± standard error. Significance was defined as *p* < 0.05.

**Table 1:** Behavioral analysis categories.

	Categories	Definition
(1)	Sniffing time	Time taken to keep the nose close and move the nostrils for each stimulus condition (type and position)
(2)	Nostril-use time	Time taken to keep the nose close and move the right (or left) nostril for each stimulus condition (type and position)
(3)	Number of nostril-uses	Number of times the right (or left) nostril was kept close to the tubes and moved for each stimulus condition (type and position)
(4)	Laterality index of nostril-use time	Data from applying behavior category (2) to the equation (right − left/ right + left)
(5)	Laterality index of the number of nostril-uses	Data from applying behavior category (3) to the equation (right − left/ right + left)
(6)	Number of times the left and right sides of the face were rubbed	Number of times the side of the face with that nostril side were rubbed against the tube after using the left or right nostrils
(7)	Number of sniffing repetitions	Number of times each stimulus condition was sniffed (type and location)
(8)	Number of trials with the longest sniff	Number of trials in which each stimulus condition (type and location) was sniffed the longest out of all trials
(9)	Number of trials first sniffed	Number of trials in which each stimulus condition (type and location) was sniffed first out of all trials

## Results

Three trials were conducted with each test subject, and a total of 90 video recordings were collected. Eight of these videos were excluded from the data analysis because of their low brightness; therefore, a total of 82 videos were analyzed.

### Duration of olfactory uptake by cats

**Fig 2:**
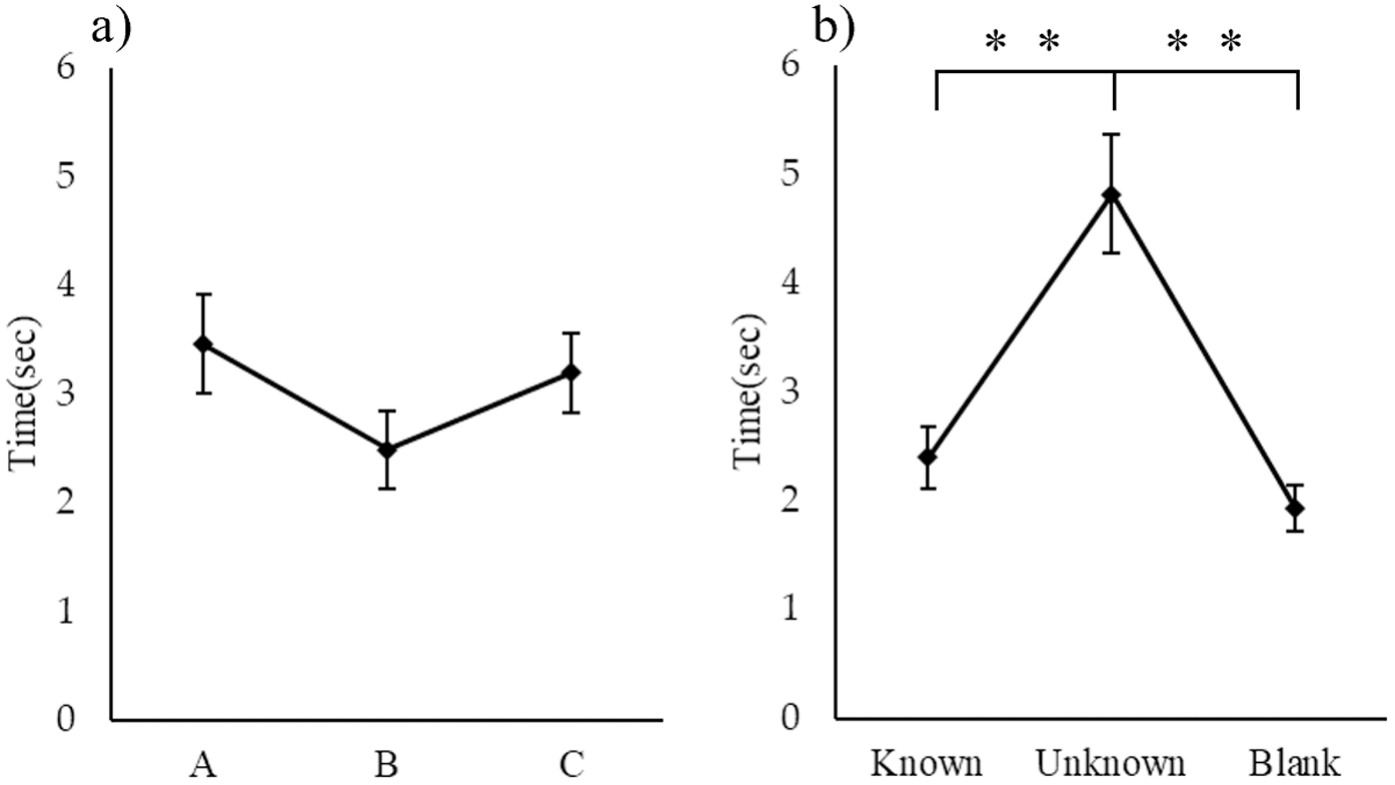
Comparison of time spent sniffing stimulus conditions (position and type) (Friedman test). a) Comparison between presentation positions, n.s. b) Comparison between types, ** *p *< 0.01.

There was no marked difference in the time that a cat spent sniffing a tube when comparing it with the presented locations (A, B, or C). However, among the three odor conditions, cats spent a longer time sniffing an unknown odor (4.82 ± 0.54 s) than sniffing either a known odor (2.40 ± 0.28 s) or blank stimulus (1.93 ± 0.21 s) (Friedman test: *p* < 0.01; Fig 2).

### Nostril use for different odor types

**Fig 3:**
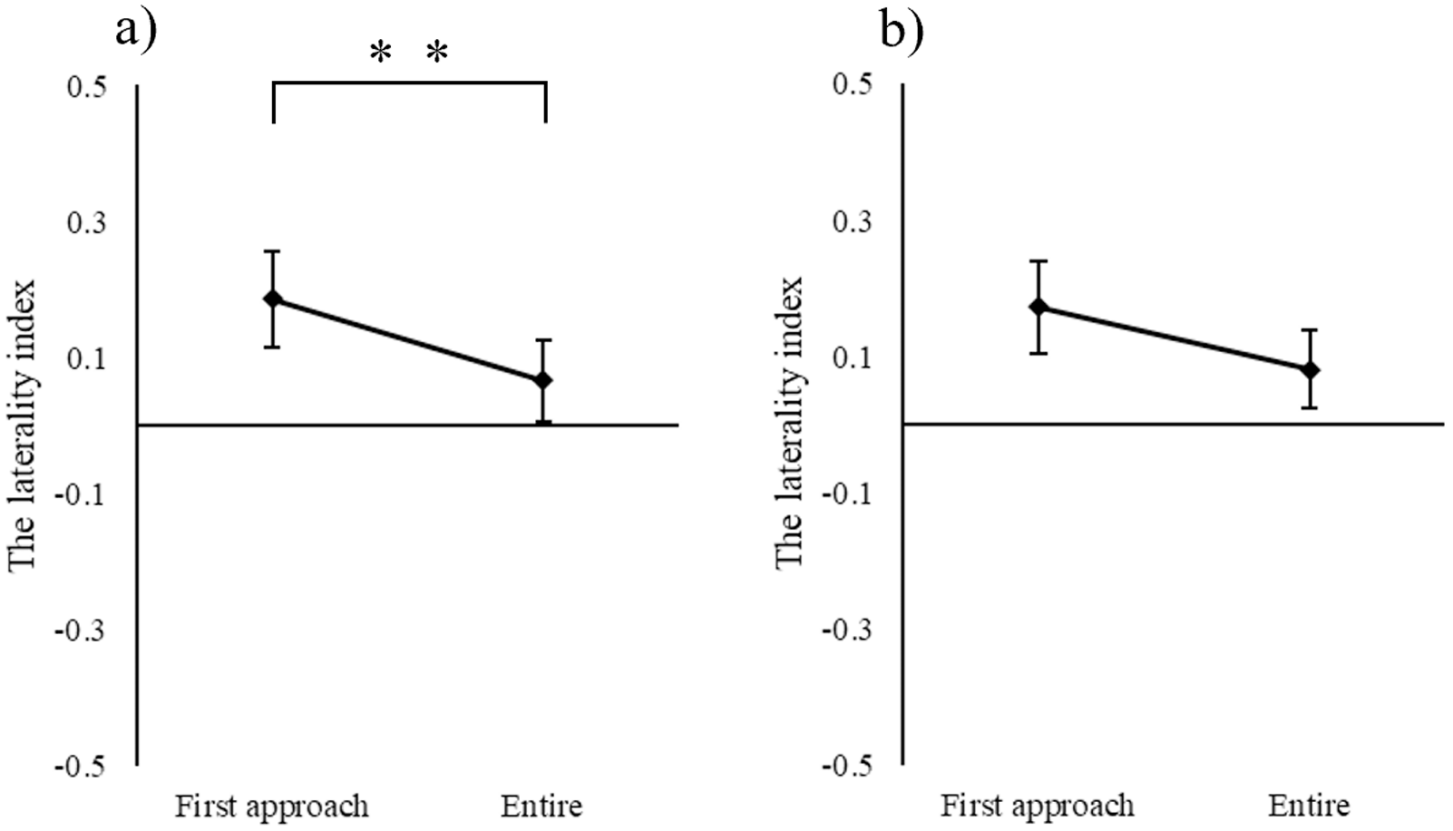
Comparison of laterality index of nostrils used for unknown odors between the first approach and overall exploratory behavior (Wilcoxon test). Laterality index of (a) time spent using nostrils, ** *p* < 0.01, b) number of nostril uses, n.s.

Negative correlations were found between the total number of sniffing repetitions and the laterality index of nostril-use time or number of nostril-uses for unknown odors (S2 Table, (1) and (2)). Therefore, the researcher calculated the laterality index based on the duration and number of times that the left and right nostrils were used for an unknown odor on first contact to it (right nostril − left/right nostrils + left nostril). The laterality index in the first approach was observed to be higher than that in the exploratory behavior, only in terms of duration (Wilcoxon test: p < 0.01; Fig 3).

### Nostril use for different odor locations

**Fig 4:**
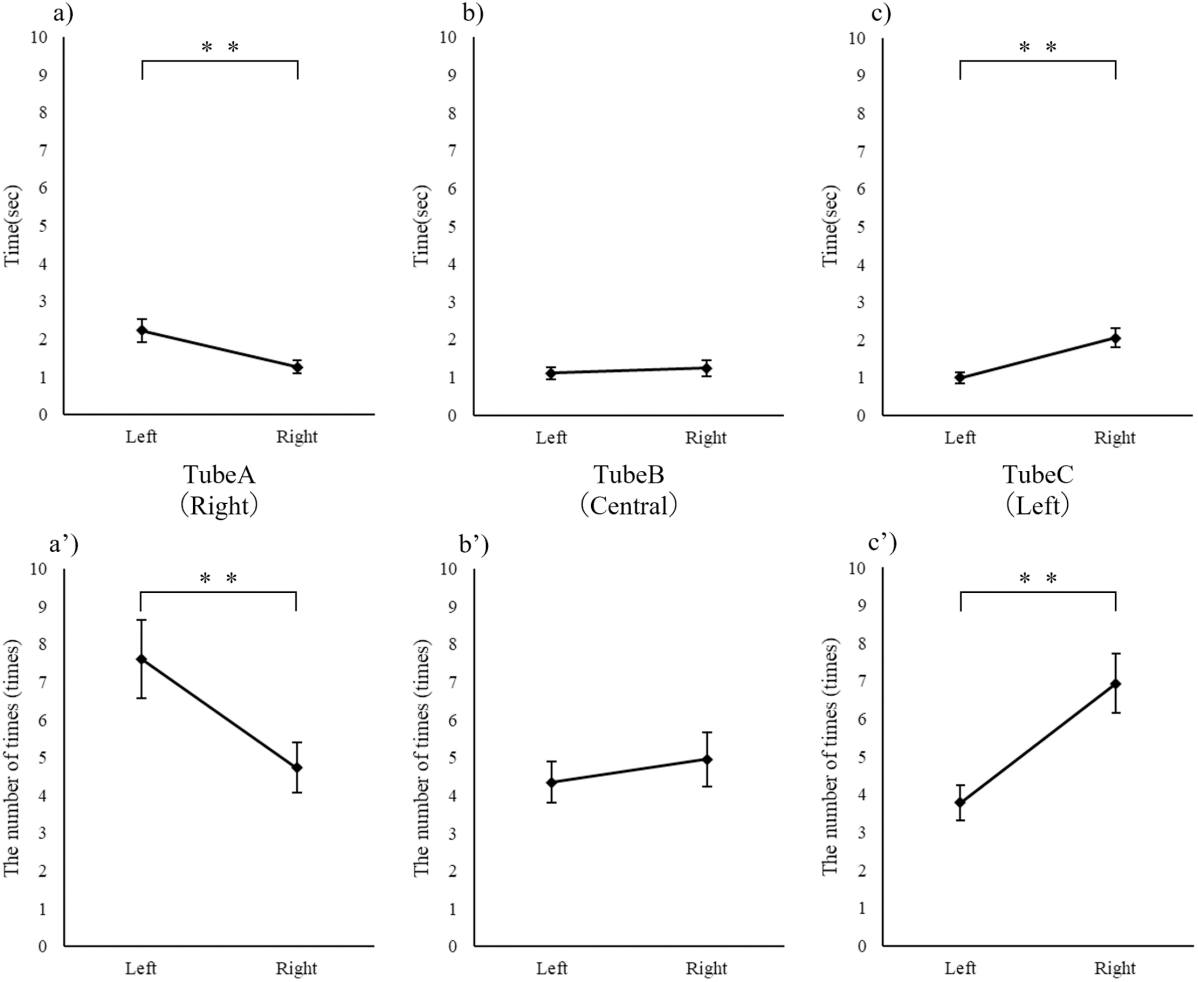
Comparison of the time (a, b, c) and number of times (a’, b’, c’) that the left and right nostrils were used at each odor stimulus position (Wilcoxon test). ** *p* < 0.01.

The duration and frequency of the left and right nostril-use to sniff tubes A, B, and C were compared. The results showed that tube A (on the right-hand side of the cat) was sniffed more often with the left nostril, whereas tube C (on the left) was sniffed more often using the right nostril. There were no differences in either the duration or frequency of nostril-use for tube B (in the center) between the right and left nostrils (Fig 4).

The incidence of cats rubbing the same sides of their faces as the nostrils used on the trial tubes was also compared, distinguishing between the right and left nostrils used for tubes A and C. The left side of the face was rubbed more frequently (21.69 ± 4.73%) than the right side (1.22 ± 1.22%) on tube A (on the right) (Wilcoxon test: *p* < 0.01). The right side of the face was rubbed more frequently (19.23 ± 4.11%) than the left side (0.41 ± 0.41%) on tube C (on the left) (Wilcoxon test: *p* < 0.001; Holm-corrected significance level, *p* < 0.005).

When associating the incidence of cats rubbing the contralateral side of their faces on tube C with the personality and relationship scales of their owner, we observed possibility of positive correlation with the impulsiveness score and a strong correlation with closeness score between the owner and the cat (S2 Table; (3) *p* < 0.05; (4) *p* < 0.005; Holm-corrected significance level, (3) *p* < 0.0071, (4) *p* < 0.0063). Therefore, the association between the incidence of rubbing of the contralateral aspect of the face when tube C contained a known or unknown odor, or blank stimulus; the impulsiveness score; and the closeness score between the owner and cat was examined. Strong positive correlations were observed between the group in which tube C contained a blank control and impulsiveness score, and between the group in which tube C contained a known odor and the cat–owner closeness scores (S2 Table, (5) and (6)).

### Relationship between the number of repeated sniffs and personality

No correlations were observed between the duration of odor sniffing and feline personality. However, a strong correlation was reported between the number of sniffing repetitions for a given odor type and cat personality (S2 Table, (7)–(26)). The results were then analyzed separately for male and female cats. In the males, a strong correlation was discovered between the number of sniffing repetitions and personality (S2 Table, (27)–(46)); however, no strong correlations were observed among these parameters for female cats.

### Relationship between the odor type sniffed first and personality

All cats were divided into three groups: those who smelled a known odor first, those who smelled an unknown odor first, and those who smelled a blank tube first. Personality scores were compared between these three groups. The group that smelled the blank tube first (59.92 ± 3.46 points) possessed a higher neuroticism score than the group that smelled the known (47.82 ± 2.69 points) odor first (Kruskal–Wallis test: *p* < 0.05). In contrast, the group that smelled the known (71.29 ± 1.53 points) odor first exhibited higher extraversion scores than the group that smelled the blank (61.83 ± 2.09 points) tube first (Kruskal–Wallis test: *p* < 0.01). Furthermore, cats in the group that smelled the known (58.71 ± 1.98 points) or unknown (59.17 ± 1.73 points) odors first had higher agreeableness scores than those in the group that smelled the blank tube first (50.21 ± 2.29 points) (Kruskal–Wallis test: *p* < 0.01).

## Discussion

### Human discrimination and lateralization in feline olfaction

The duration for which the cats sniffed the odor stimuli at different presentation positions were similar, confirming that they did not have a preference for the order in which odors were sniffed. However, cats spent a substantially longer time sniffing the odor of an unknown person than that of their owners. Cats are known to respond differently to familiar and unfamiliar people [12]. Weaned kittens sniff unknown female cats longer than they sniff their mothers [10]. During habituation and de-habituation trials, cats alter their responses to known and unknown stimuli [11,12]. Our results suggest that they may be able to discriminate between known and unknown humans based on their odor.

When exposed to odors of an unknown person, cats initially used their right nostril more frequently; however, after repeated sniffing, they shifted to using their left nostril more frequently. Dogs exhibit similar behavior when sniffing novel, non-narcotic, non-aversive stimuli (food, lemon, vaginal discharge, and cotton swabs) [15]. Horses also preferentially use their right nostril when sniffing feces, arousing odors, or novel odors [19–22]. Cats preferentially use the right nostril for the smell of humans in a state of fear [14]. Our study also confirms that cats display the lateralized use of their left and right nostrils in response to unfamiliar odors. Studies have suggested that not only dogs but also fish, birds, and other vertebrates process novel information using the right brain hemisphere, which is then habituated and classified; the left brain is responsible for further behavior when a routine response emerges [15]. Aggressive responses in several vertebrate species are controlled by the right hemisphere of the brain, whereas the left hemisphere suppresses the expression of aggression in domestic chicks [23]. The cats in the current study may have used their right nostrils more frequently during initial olfactory exploration because of experiencing a potential alarm and fear of novel odors. Moreover, their continued exploratory behavior via the olfactory organs may have led to a shift in use of the left nostril as the cats classified the stimuli and acclimated to them over time. These results cannot indicate whether cats recognize specific persons from olfactory cues. This theory warrants further validation using a cross-modal paradigm.

### Use of different nostrils in relation to the tube location

**Fig 5:**
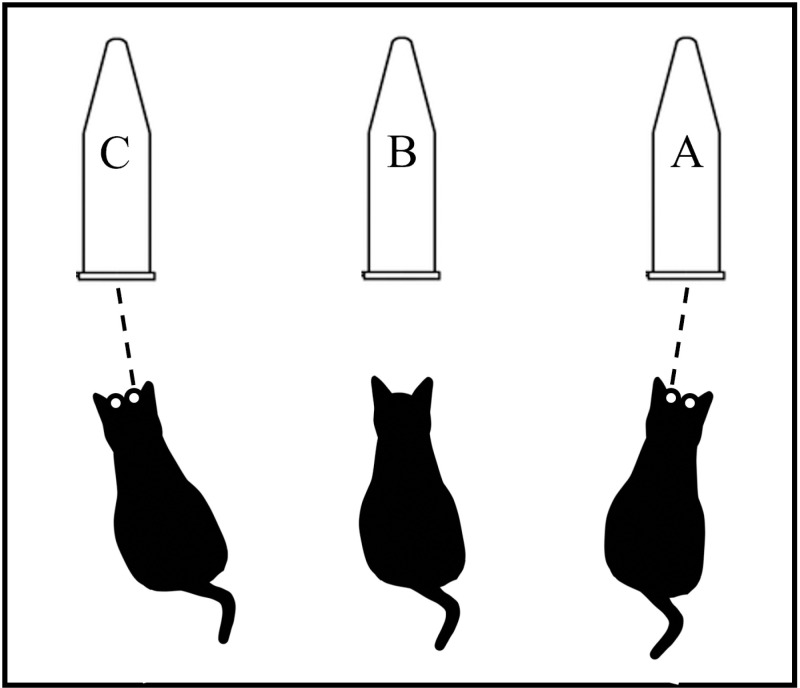
Schematic diagram of nostril use for odor stimulus presentation position. The left nostril is contralateral to tube A, and the right nostril is contralateral to tube C.

**Fig 6:**
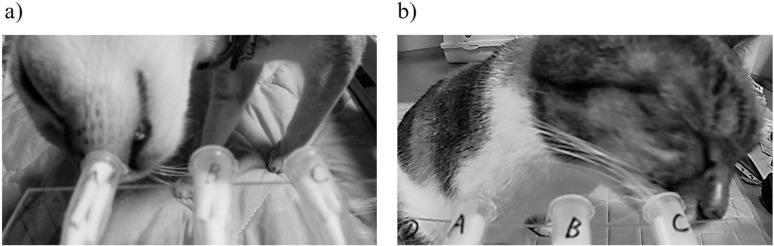
Photographs of cats. a) Sniffing the tube on the left and right side with a tilted head. b) Rubbing the same side of the face as the nostril that was used immediately prior to this.

The cats exhibited exploratory behavior, mainly sniffing using their contralateral nostrils in relation to odor-presenting tubes located on their left and right sides. No marked difference was observed in the time spent using the left and right nostrils to sniff the central tube (Fig 5). Two hypotheses may explain such behavior. The first hypothesis is that the cats align their left and right nostrils vertically to allow the uptake of odor molecules from only a specific tube into one nostril, considering that the three odor stimulus tubes were arranged horizontally at a relatively narrow interval of 3 cm (Fig 6a). The second hypothesis is that the cats may have selected the use of their contralateral nostrils based on the assumption that they would rub their bodies against the odor. The incidence of cats sniffing the outer stimulus tubes with their contralateral nostrils followed by rubbing the contralateral aspect of their faces against the tubes was substantially greater than the instances in which they sniffed these tubes with their ipsilateral nostrils before rubbing against them with the ipsilateral side of their faces. Cats routinely rub their faces and other body parts against objects during marking behavior [24]. Therefore, we could infer that sniffing behavior may be coupled with the acquisition of the correct bodily orientation to allow subsequent rubbing behavior for marking purposes (the central tube B is in the way of using the ipsilateral side for rubbing) (Fig 6b).

One form of head rubbing behavior in cats, allorubbing, is performed on social partners as well as humans [25,26]. In our study, a strong, positive correlation was observed between the incidence of marking behavior toward a blank tube C and the impulsiveness score of cats, and toward tube C containing the odor of the owner and cat–owner intimacy scores. These results suggest that marking behavior accompanied by olfactory exploration consists of a series of habitual behaviors, whereas rubbing the owners could be a way of showing affection. However, since the owner of the cat presented the odor conditions in our study, it was not possible to discern whether the cats directed their rubbing behavior at the odors or at the owners. Future research should consider the type of motivation that stimulates rubbing behavior toward odors. In addition, face-rubbing behavior includes not only allorubbing, which is performed as an affinity behavior, but rubbing is a marking behavior and for rubbing against odors [8,27]. The definition of the behaviors observed in this study should be examined in future studies.

### Relationships between feline personality, sex, and odor-sniffing behavior

There was a strong association between repeated sniffing behavior and feline personality traits. A strong association was observed between sniffing and neuroticism and agreeableness scores in males, but not in females. This finding suggests that the association is more pronounced in males. In fish, males have been recorded as behaving more boldly than females [28–30]. Our study found no association between the duration of olfactory exploration and personality. However, among males, individuals with higher neuroticism scores sniffed the stimulus tube more busily (i.e., cats sniffed different tubes more often during exploratory behavior), whereas those with higher agreeableness scores sniffed the tube more calmly (i.e., the number of approaches to the tube during exploratory behavior was minimal). Thus, in cats, personality type may influence behavioral responses to odor stimuli in males. To obtain male and female responses in future studies, the sample size should be increased for optimal replication.

We observed an association between feline personality and the choice of odor type being sniffed first (known, unknown, or blank). This association suggests that, in cats, a specific process may be responsible for the decision of which odor to sniff first before actively approaching and sniffing the olfactory stimulus. In other words, the cat may have been able to perceive volatile odor molecules and discriminate between tubes containing human odor and blank tubes even before it put its nose close to the tube. Such a process may be responsible for certain personality features. The existence of such a process and its associated behavior warrants further investigation.

Although feline personality was associated with odor-sniffing behavior, no specific association existed between cat–owner relationships and exploratory olfactory behavior. The relationship of cats with their owners was related to the number of years they have been together and to emotional closeness as per the CORS scale. However, no association was found between factors that could potentially influence the experience of a cat with odor exposure and the actual perception of odors from the owner. This suggests that a series of exploratory olfactory behaviors may be an intrinsic feature of cats and is not influenced by experience or learning. Future studies need to examine, from various perspectives, whether the relationship between an owner and a cat influences the social cognition via olfaction in this animal.

## Conclusions

In this study, we described the behavioral responses of *Felis catus* to human olfactory stimuli. We suggest that cats use their olfaction for the recognition of humans. In addition, we suggest that olfactory exploratory behavior in cats is related to personality features and that there is lateralization of the left and right nostrils in the detection of odors. This lateralization may have been influenced by the differences in brain hemisphere function. Lastly, we record characteristic rubbing (marking) behavior occurring after sniffing, indicating that sniffing may be an exploratory behavior preceding the rubbing of odor (marking) in cats.

## Supporting information

S1 Appendix Questionnaire items. (XLSX)

S2 Table Items for which correlations were found. (XLSX)
